# Supplementary material for: Effect of pulse-current-based protocols on the lithium dendrite formation and evolution in all-solid-state batteries
Source: Nat Commun. 2023 Apr 27;14:2432. doi: 10.1038/s41467-023-37476-y (PMC10140044; doi:10.1038/s41467-023-37476-y)
Supplement: Supplementary file 1 — Supplementary Information [file 41467_2023_37476_MOESM1_ESM.docx]

Supplementary Information

**Effect of pulse-current-based protocols on the lithium dendrite formation and evolution in all-solid-state batteries**

V. Reisecker^a,b#^, F. Flatscher^b,c#^, L. Porz^c^, C. Fincher^d^, J. Todt^e^, I. Hanghofer^f^, V. Hennige^f^, M. Linares-Moreau^g^, P. Falcaro^g^, S. Ganschow^h^, S. Wenner^i^, Y.-M. Chiang^d^, J. Keckes^e^, J. Fleig^j^, D. Rettenwander^a,b,c*^

^a^ Institute of Chemistry and Technology of Materials, Graz University of Technology, Graz, Austria.

^b^ Christian Doppler Laboratory for Solid-State Batteries, NTNU Norwegian University of Science and Technology, Trondheim, Norway.

^c^ Department of Material Science and Engineering, NTNU Norwegian University of Science and Technology, Trondheim, Norway.

^d^ Department of Materials Science & Engineering, Massachusetts Institute of Technology, Cambridge, MA, USA
^e^ Department of Materials Physics, Montanuniversität Leoben and Erich Schmid Institute for Materials Science, Austrian Academy of Sciences, Leoben 8700, Austria

^f^ AVL List GmbH, Graz, Austria

^g^ Institute of Physical and Theoretical Chemistry, Graz University of Technology, Graz, Austria

^h^ Leibniz-Institut für Kristallzüchtung, Berlin, Germany

^i^ Sintef Industry, Department of Materials and Nanotechnology, Trondheim, Norway
^j^ Institute of Chemical Technologies and Analytics, TU Wien, Vienna, Austria

^#^Authors, V. Reisecker and F. Flatscher, equally contributed to this work.

^*^Corresponding author: [daniel.rettenwander@ntnu.no](mailto:daniel.rettenwander@ntnu.no)


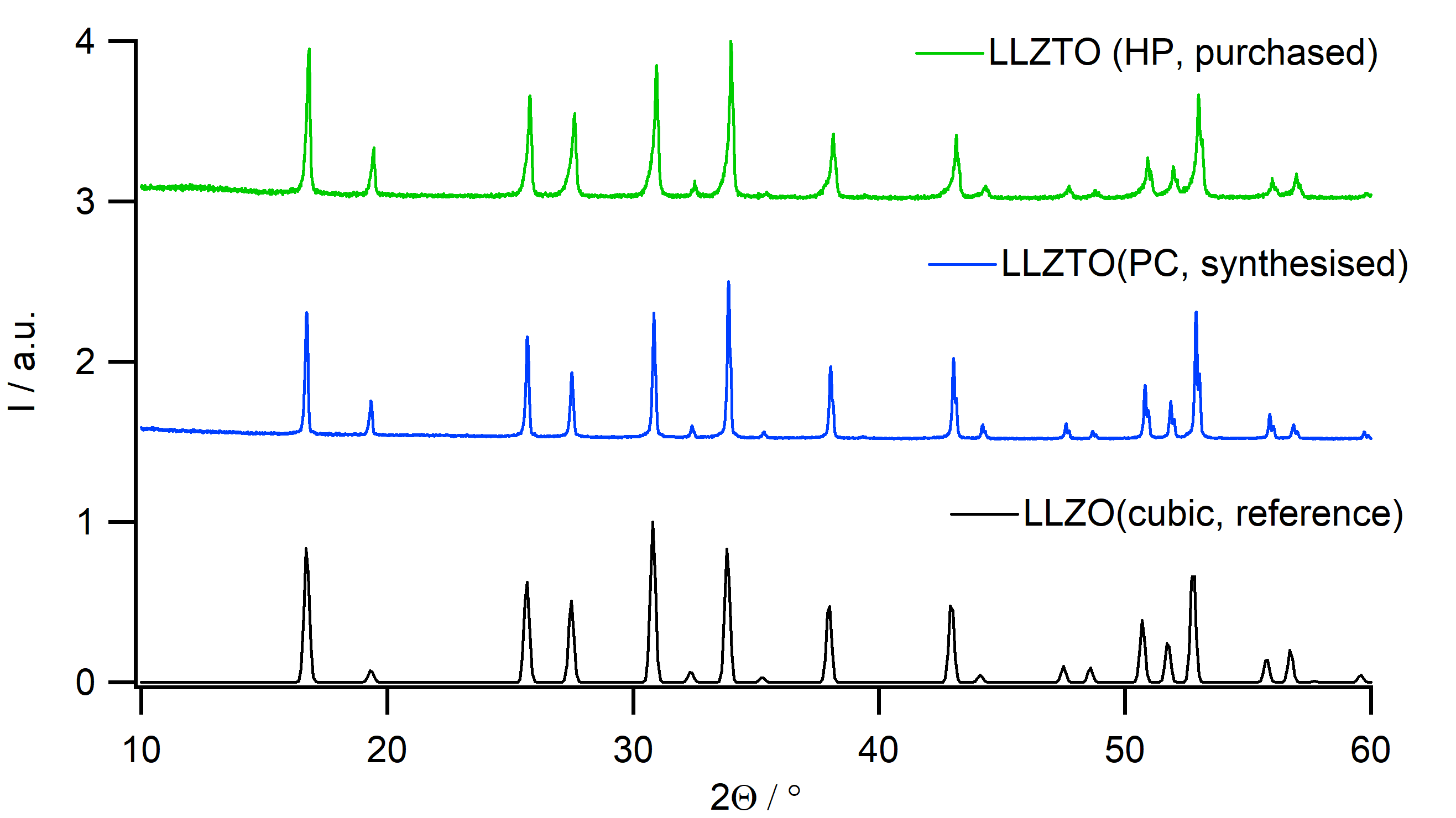


**Supplementary Fig. 1:** **XRD data of polycrystalline samples**. Obtained X-Ray Diffraction patterns of polycrystalline Li_6.5_La_3_Zr_1.5_Ta_0.5_O_12_ synthesized via solid-state route (PC, blue) and polycrystalline hot-pressed (HP, green) Li_6.4_La_3_Zr_1.4_Ta_0.6_O_12_ in comparison to cubic Li_7_La_3_Zr_2_O_12_ from ICSD (#422259, black).


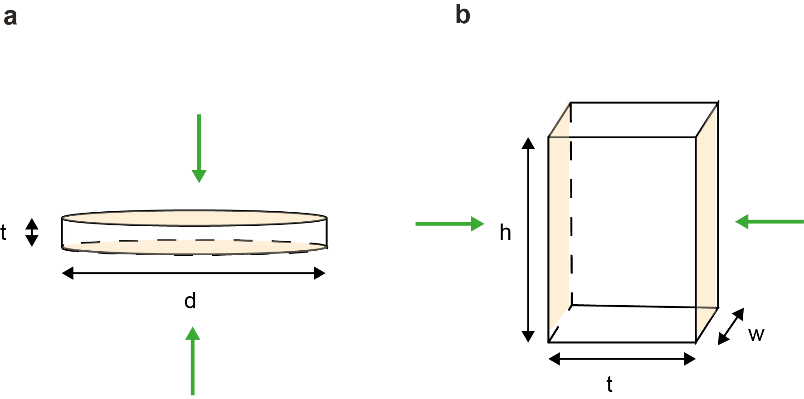


**Supplementary Fig. 2:** **Geometries of samples.** Schematics illustrating a typical cylindrical PC pellet (a) and a SC/HP cuboid (b). The polished and heat-treated surface areas are highlighted in orange, which in the end were coated with a molten Li:Sn alloy as indicated by the green arrows.

**Supplementary Fig. 3:** **AFM results of samples.** Atomic Force Microscopy analyses were conducted to assess the roughness and defect concentration of the SC (a) and HP (b) surfaces. Both topography images reveal scratches on the polished electrolyte surface. The depth of these scratches is, however, lower for the SC (a) than for the HP (b) pellet, denoting a lower surface roughness (see height profiles in Figure 1c). Average RMS surface roughness was calculated from these measurements and resulted in (3 ± 1) nm and (8 ± 3) nm, for SC and HP respectively.


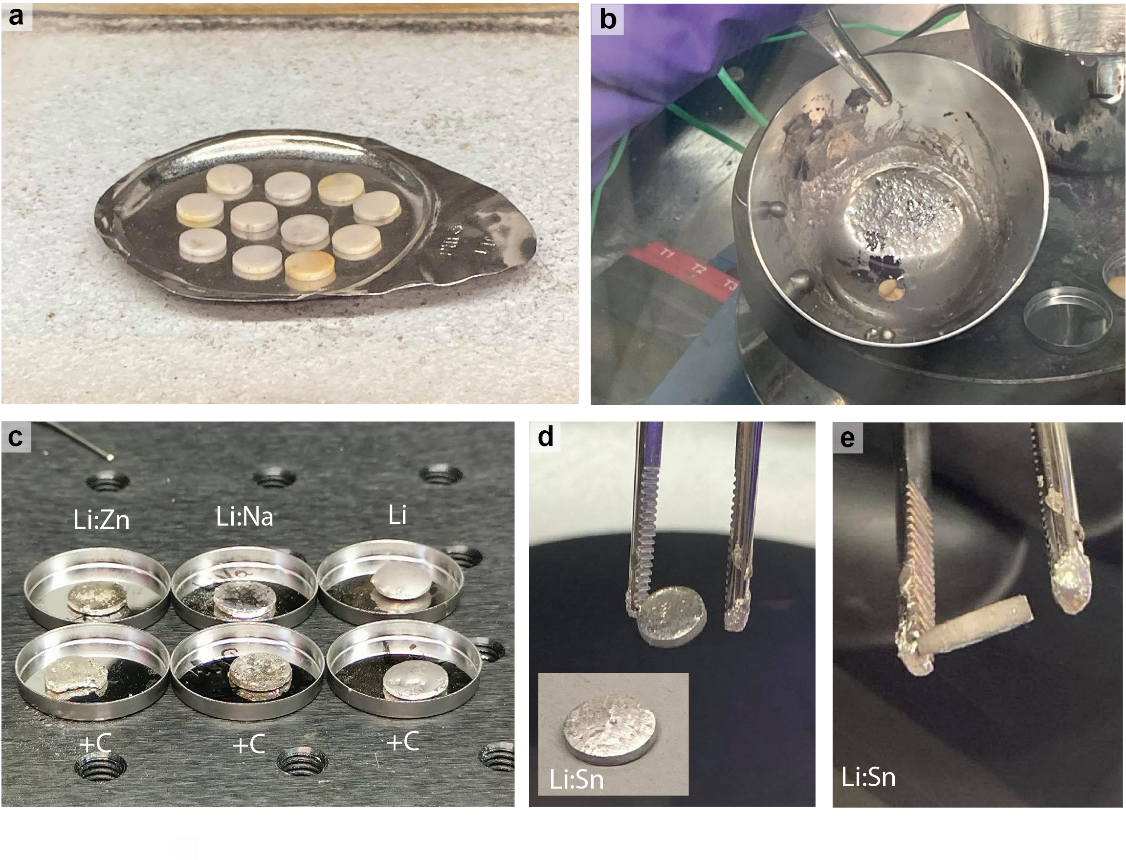


**Supplementary Fig. 4: Coating approaches of pellets. (**a) finished PC samples after the polishing and heat treatment. (b) PC pellet half immersed in the Li:Sn alloy when heated up to 250 °C in a Pt-crucible. (c) Comparison of the Li:alloy wetting capabilities of some different coating approaches tested. The Li/Zn alloy contained 5mol% Zn and the Li/Na alloy 21.4mol% Na, +C denotes the use of a carbon interlayer. (d,e) Images of a PC pellet completely coated with the Li:Sn alloy.


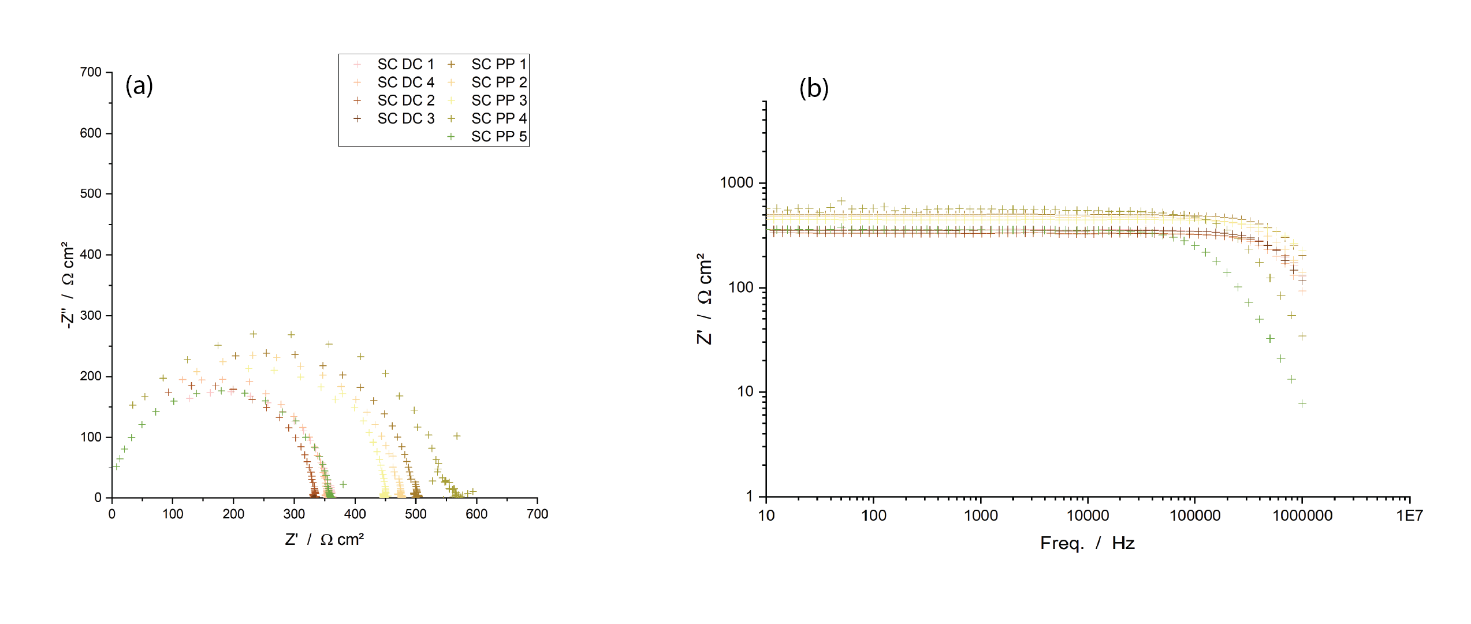
**Supplementary Fig. 5: EIS data of SC samples.** (a) Impedance data of the SC samples in a Li|SC|Li cell measured at 21±1°C measured prior to direct current (DC) or pulse plating (PP) experiments along with the respective (b) Bode plots.


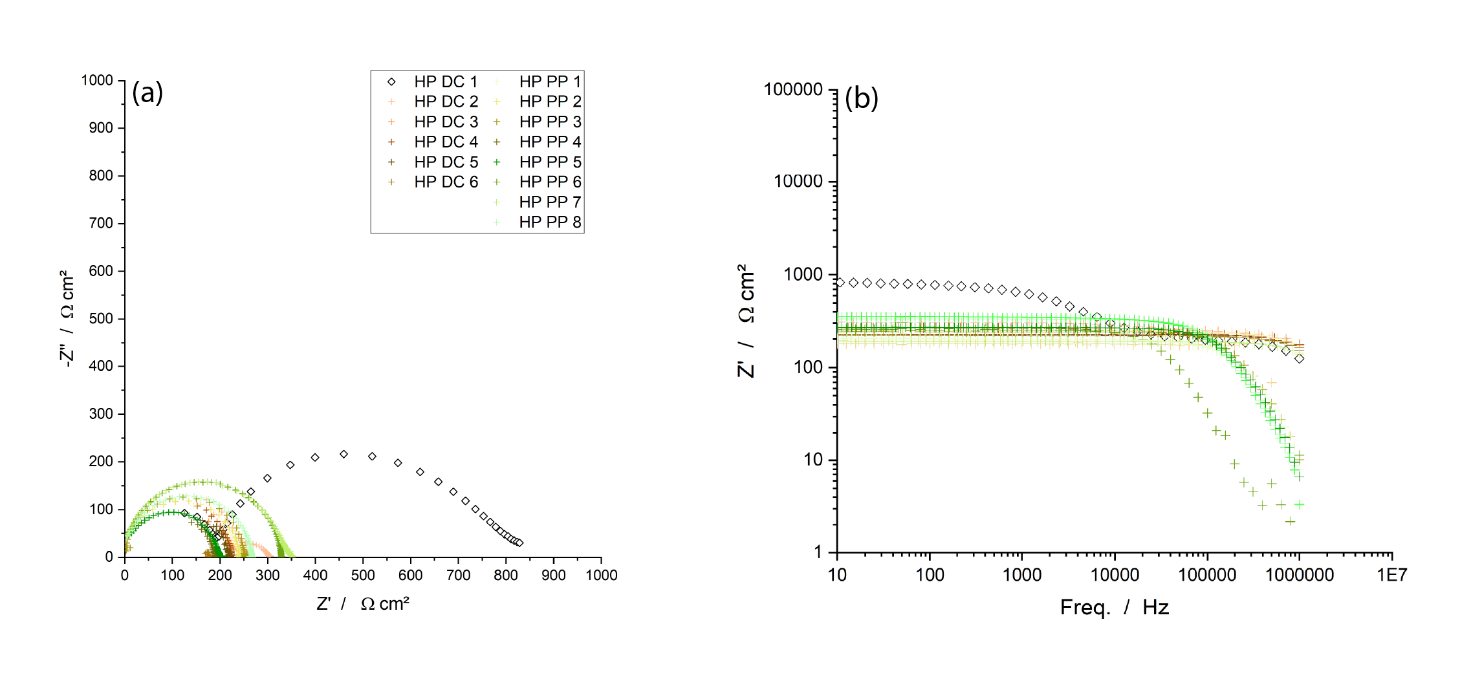


**Supplementary Fig. 6: EIS data of HP samples.** (a) Impedance data of the HP samples in a Li|HP|Li cell measured at 21±1°C prior to direct current (DC) or pulse plating (PP) experiments along with the respective (b) Bode plots. HP DC 1 (black marker) represents a non-heat-treated sample and HP DC 2 did not undergo a proper polishing sequence which demonstrates the importance of a proper sample treatment prior to coating.


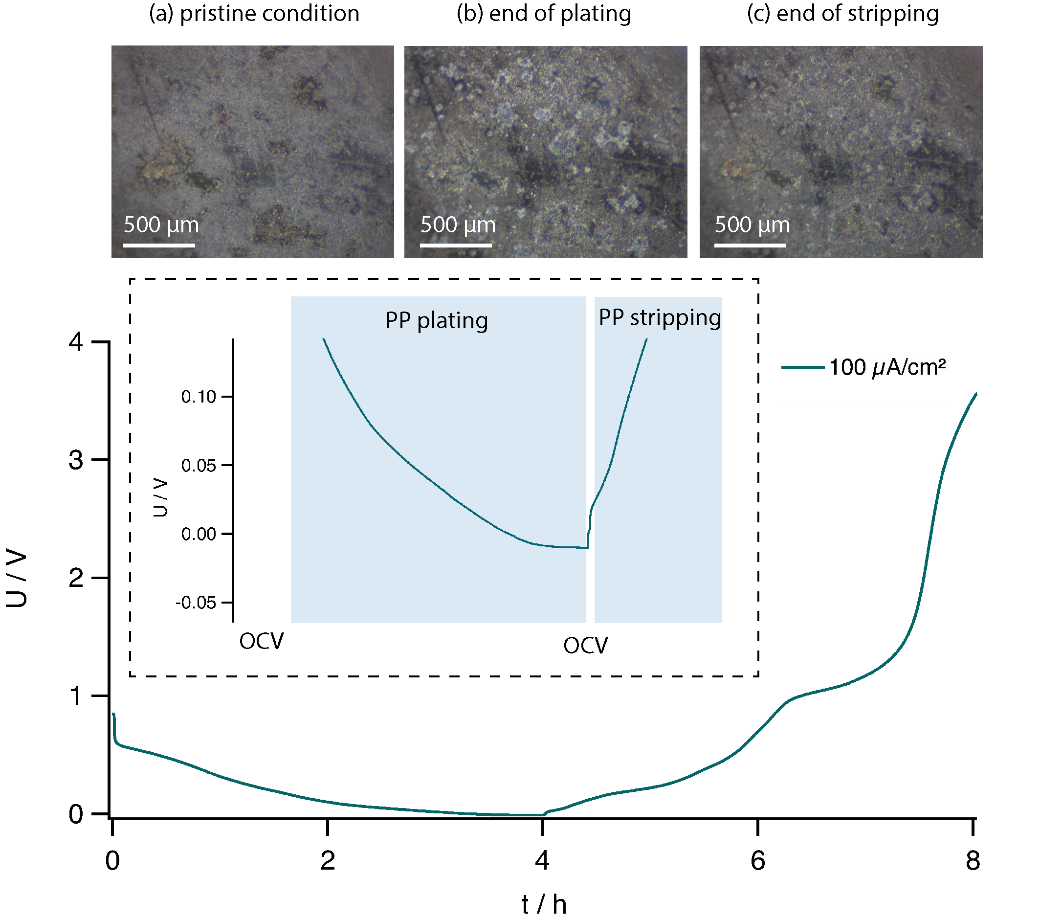


**Supplementary Fig. 7: Plating experiments of polycrystalline samples.** Plating profile of a PC sample coated with Au on one side and Li:Sn on the other, in a Li|PC|Au cell treated at 1:1 µs pulse pause (PP) ratio at 100 μA/cm^2^ at 21±1°C.The close-up inspection window shows the switch from the plating to the stripping step and the open circuit voltage (OCV) in between. In situ Optical Microscopy images on top show the surface of the Au electrode in its pristine condition (a) and confirm Li is being plated at the opposing Au electrode (b) and stripped again in the subsequent half cycle (c).


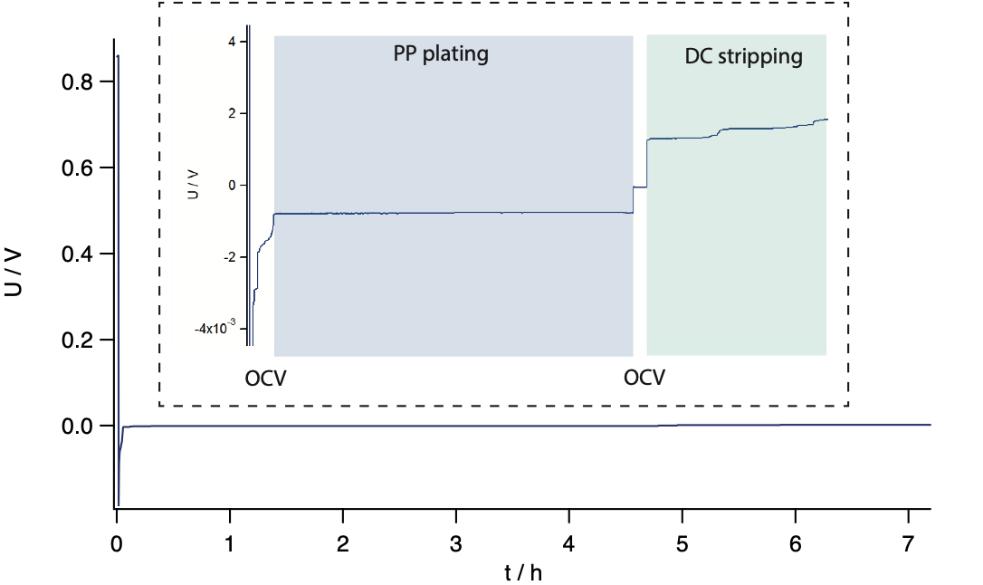


**Supplementary Fig. 8:** **Efficiency experiments of polycrystalline samples.** Voltage profile of a PC-sample in a Li|PC|Au cell undergoing an efficiency measurement where 200 µAh/cm² of lithium are plated at 50 μA/cm^2^ pulsed current application (again 1:1 µs pulse pause ratio, PP) and subsequently stripped with direct current (DC) application at 21±1°C. A close-up inspection window shows the switch from the PP plating step to the direct current stripping step and the open circuit voltage (OCV) in between.


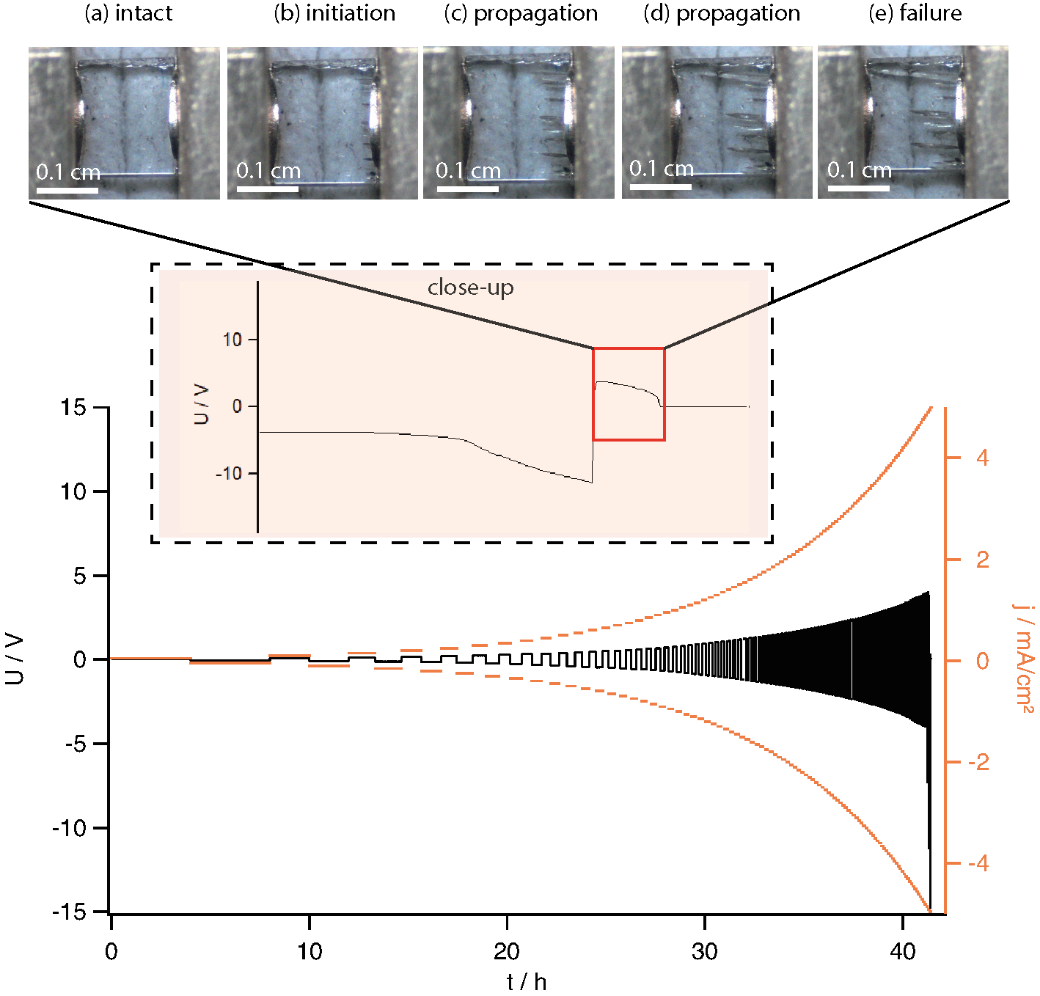


**Supplementary Fig. 9:** **Cycling performance of SC sample.** Cycling profile of the SC sample measured in a Li|SC|Li cell at 21±1°C (a, intact state) which achieved the highest effective CCD value during this study being (4.59 ± 0.05) mA/cm^2^ after application of a direct current program. A close-up inspection window and simultaneously taken in situ Optical Microscopy images of the SC at the top show failure. First, the voltage increases drastically, presumably due to contact loss from void formation. In the subsequent plating step, the voltage response drops as the lithium filaments initiate (b) and propagate through the SC (c-d) until the cell fails (e).


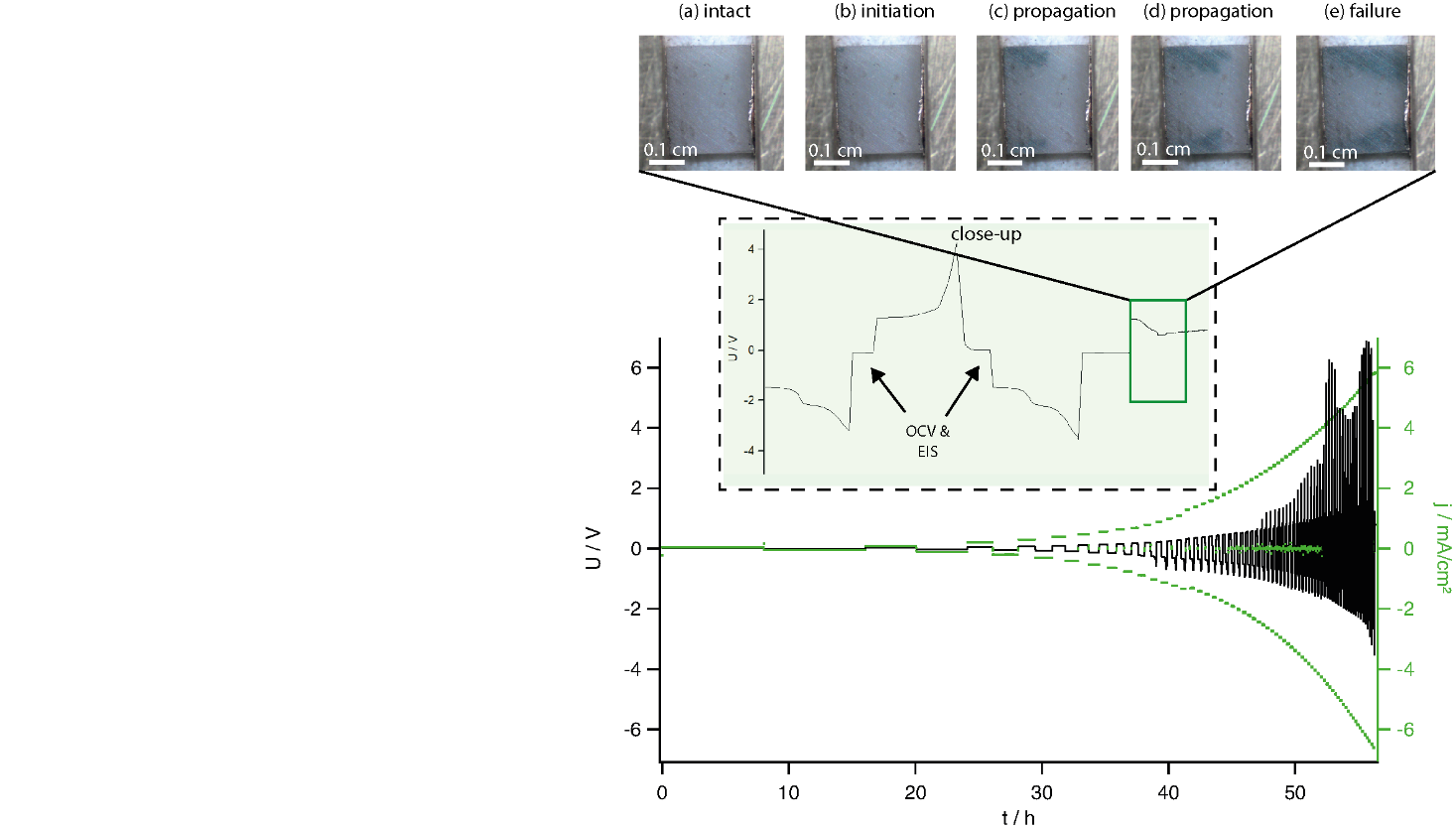


**Supplementary Fig. 10:** **Cycling performance of HP sample.** Cycling profile of the HP sample measured in a Li|HP|Li cell at 21±1°C (a, intact state) which achieved the highest CCD during this study being (6.6 ± 0.1) mA/cm^2^ after application of a 1:1 (µs) pulsed current program. In contrast to the direct current program, here, 10 mV perturbation Potentiostatic Electrochemical Impedance measurements from 1 MHz to 10 Hz with 1 minute OCV were performed in between half-cycles . Again, a close-up inspection window along with simultaneously taken in situ Optical Microscopy images of the HP at the top show the dropping voltage response as the lithium filaments initiate (b) and propagate through the HP (c-d) until the cell fails (e).


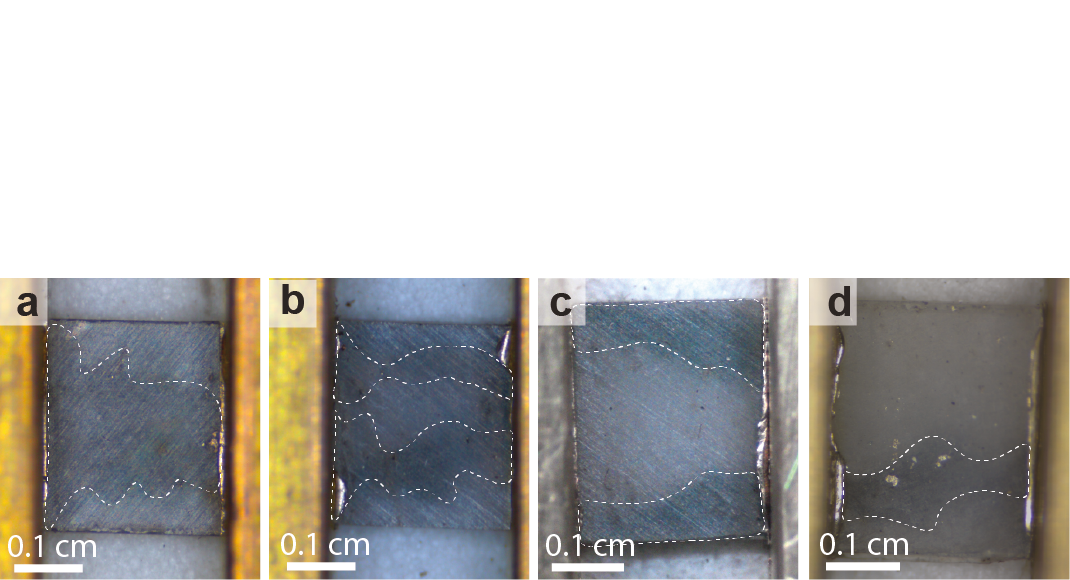


**Supplementary Fig. 11: Typical dendrite growth in HP samples.** In situ Optical Microscopy images of a selection of failed HP samples. Note that, in contrast to the SC samples, where mostly single dendrites were observed, the filaments in the HP samples rather showed an areal growth. An example of such a dendrite growth can be found in Supplementary Video 5 showing sample (c).


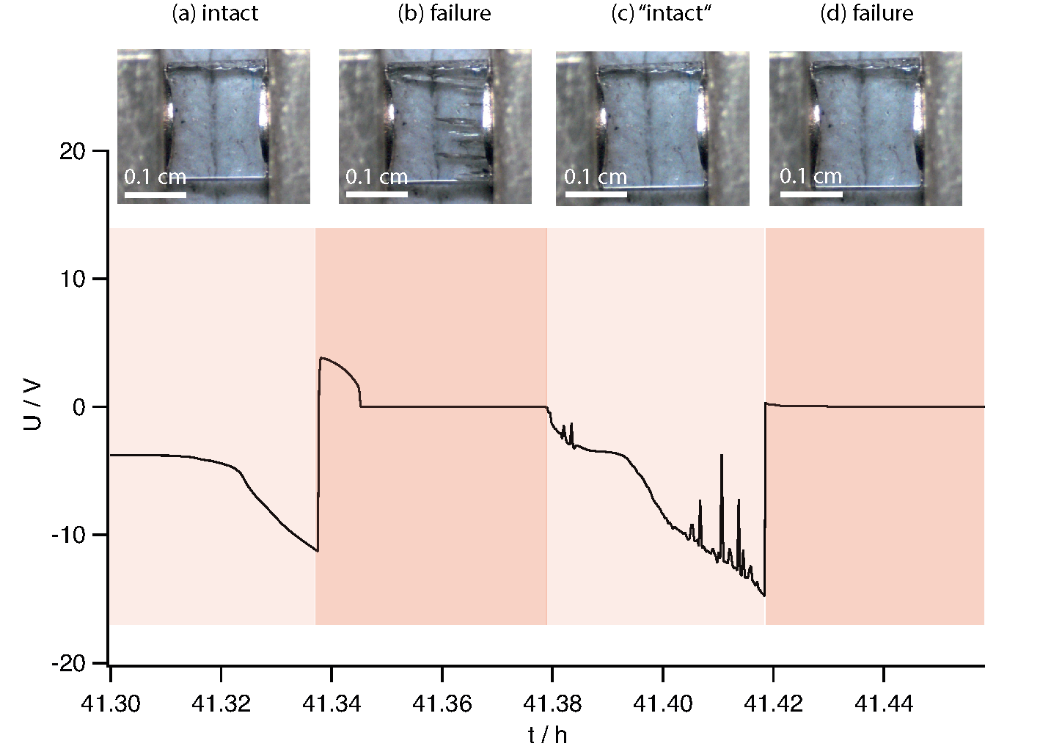


**Supplementary Fig. 12: Reversibility of Li dendrites.** Voltage profile of an SC sample, measured in a Li|SC|Li cell at 21±1°C, treated with a direct current cycling program combined with in situ Optical Microscopy images. It shows the rarely encountered peculiar behavior, that at high currents a dendrite can seemingly retract to an apparently intact sample. After short circuiting for the first time, which happens after the switch of current from (a) to (b), the lithium filaments apparently form back in the following half-cycle (c), and the sample appears to be optically and electrically intact again. In the subsequent half-cycle, a Li filament once more grows through the upper area of the SC and short-circuits the sample for good this time (d). This process can be seen in motion in Supplementary Video 4.


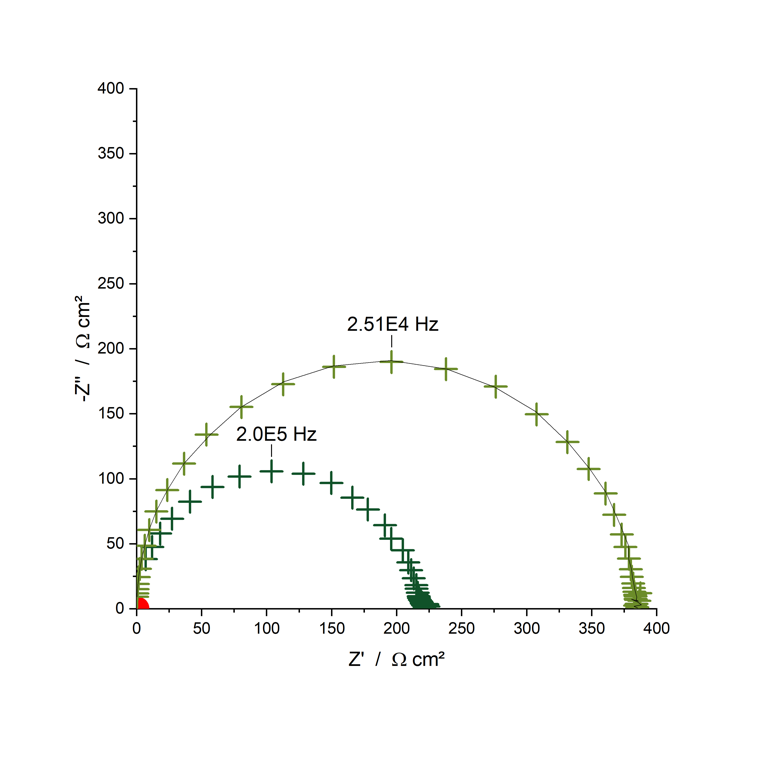


**Supplementary Fig. 13**: **Proof of Li filament reversibility.** Measured impedance responses of an SC sample in a Li|SC|Li cell at 21±1°C prior to failure (grey markers representing older cycles, green markers the last cycle prior to first failure), once short-circuited (light red) and after the Li filaments formed back completely (dark red). In the subsequent half-cycle, the sample then shorts again (light red).


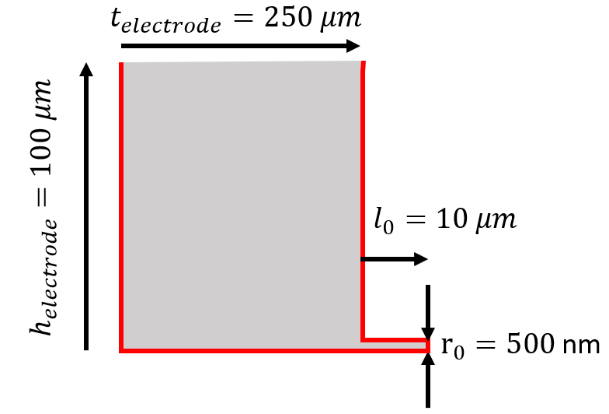


**Supplementary Fig. 14:** **COMSOL modelling of Li penetration**. Model used for the COMSOL Multiphysics finite element analysis to study the deformation of an expanding metal filament. The thickness *t_electrode_* and height *h_electrode_* of the metal electrode were set to 250 µm and 100 µm, respectively and the length of the formed metal filament *l_0_* was assumed to have a length of 10 µm. The “roller” boundary conditions are marked in red.

**
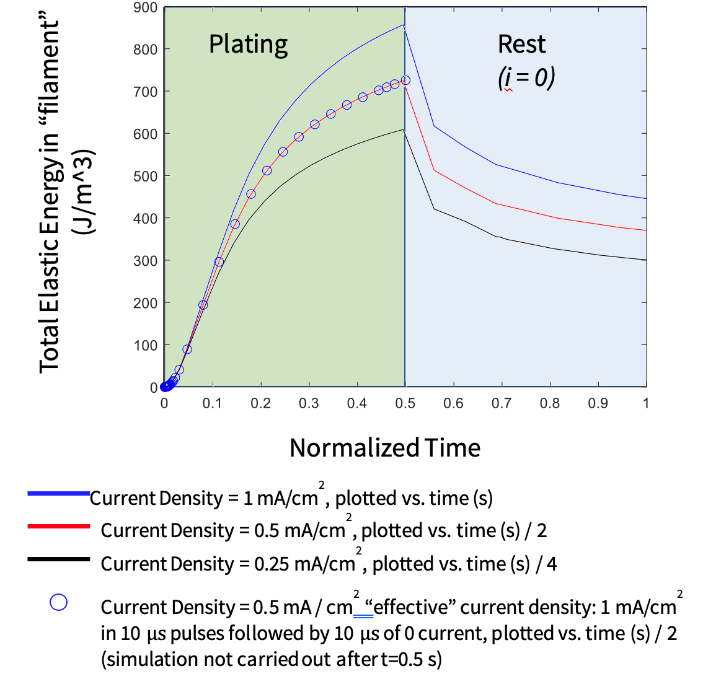
**

**Supplementary Fig. 15:** **Analysis of total elastic energy evolution.** The total elastic energy in the “filament” plotted as a function of normalized time when Li metal is being plated (green region) and when the current is switched off during a pausing time (blue region).


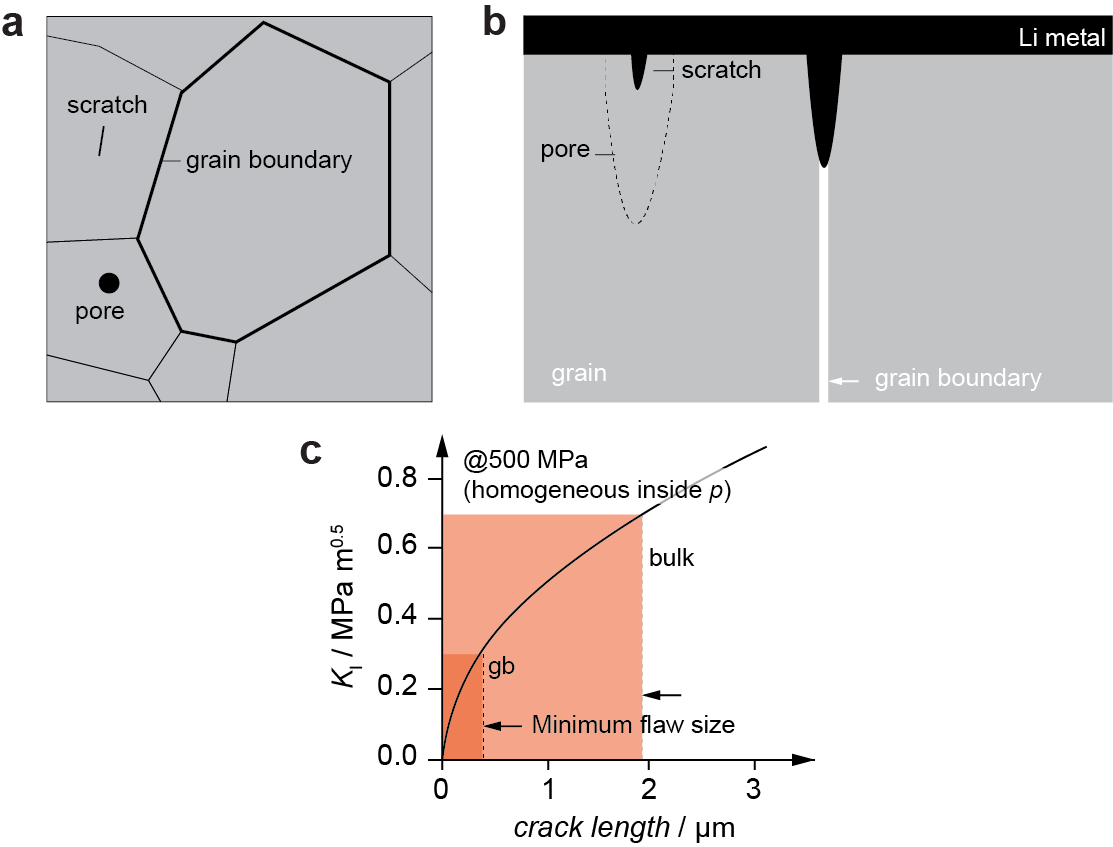


**Supplementary Fig. 16:** **Fracture stress intensity factor *K_l_* as a function of the defect length (crack).** The minimum flaw size for a grain boundary (gb) and a grain are shown for a randomly chosen critical stress intensity factor of 500 MPa, assumed as a homogeneous pressure p inside (arbitrarily chosen as the real value is not known so far).


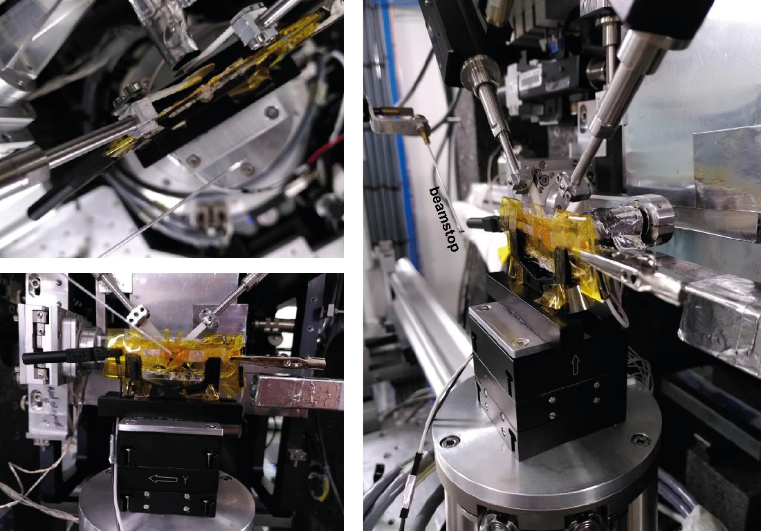

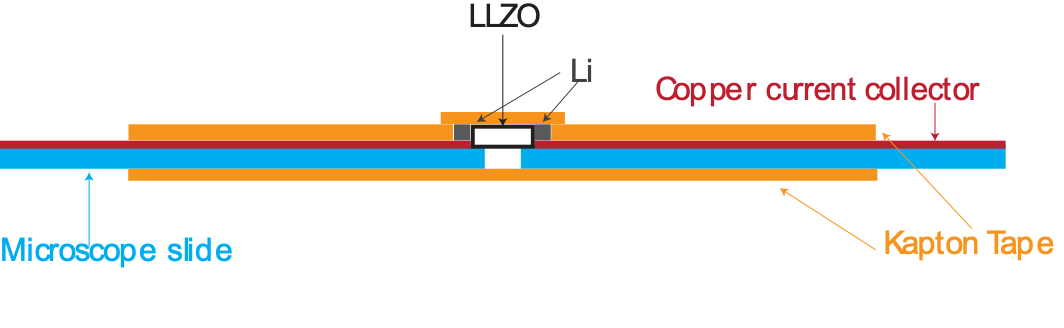


**Supplementary Fig. 17: Setup used for nanoXRD studies.** Images of the setup used for the nanoXRD study at the beamline ID13 at the ESRF. An HP sample is placed on a microscope slide coated with Li:Sn contacting a Cu current collector. A small opening below the sample helps to reduce interaction of the slide with the beam. The whole sample is encapsulated in Kapton tape to protect the Li from air and moisture. A schematic sideview of the setup is shown at the bottom. The X-ray optics are placed behind the sample in the beam path to focus the beam down to a spot size of 30 nm. The measurement was done at a temperature of 22°C.


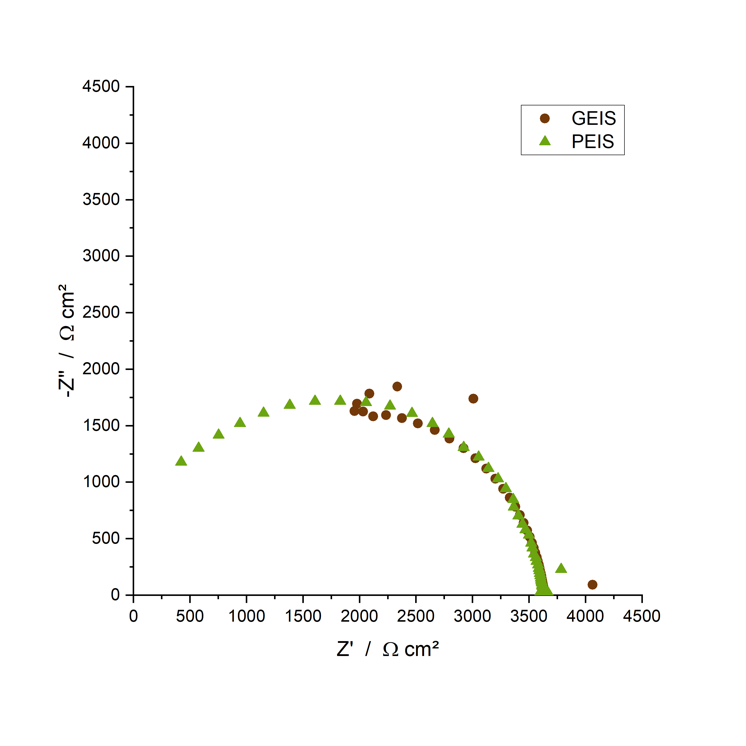


**Supplementary Fig. 18:** **EIS data comparison of GEIS and PEIS mode.** Nyquist plot comparing a 10 mV Potentiostatic Electrochemial Impedance Spectroscopy (PEIS) measurement (green) to a 1 µA Galvanostatic Electrochemical Impedance Spectroscopy (GEIS) measurement (orange) done on a Li:Sn-coated HP sample in a Li|HP|Li cell at 21±1°C. Frequencies were varied from 1 MHz to 1 Hz. Except for the inductive loop at high frequency parts for the GEIS measurement similar results are obtained.


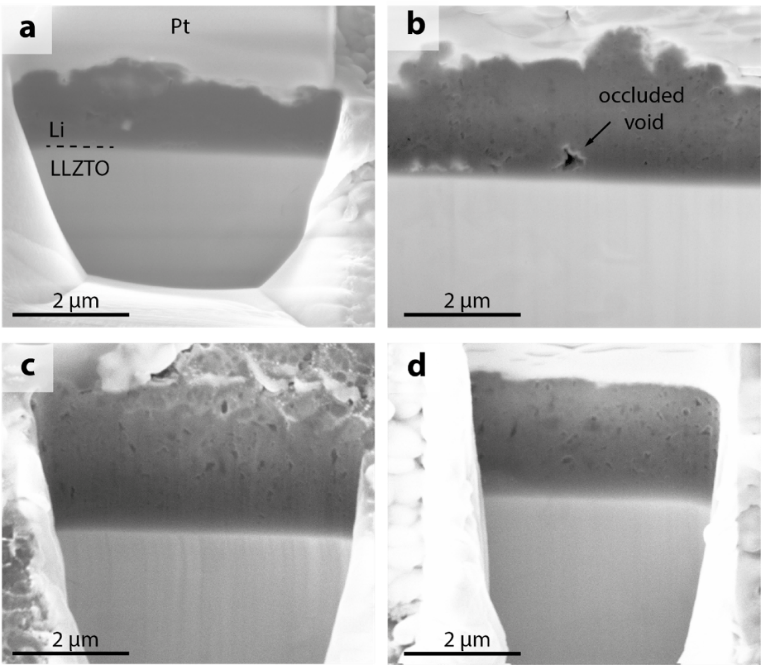


**Supplementary Fig. 19:** **SEM images of FIB cuts performed at an HP|Li interface, at two different positions.** In both cases, the Li electrode was coated afterwards with a protective Pt layer at the cut position for imaging purposes. Images were taken at 10 kV and 0.69 nA (a,b) or 0.17 nA (c,d) at a working distance of 4.1 mm. The HP sample treated with direct current is shown in subfigures a and b and was cycled until failure at 21±1°C (200 µA/cm²). Subfigures c and d show the interface of the HP sample cycled with pulsed currents until it reached the same current density as the sample treated with direct current. No significant difference becomes apparent as both interface regions show occluded voids and an intimate contact between LLZTO and Li. Therefore, it can be assumed that the deposition of Li, as expected, works smoothly with pulsed currents.


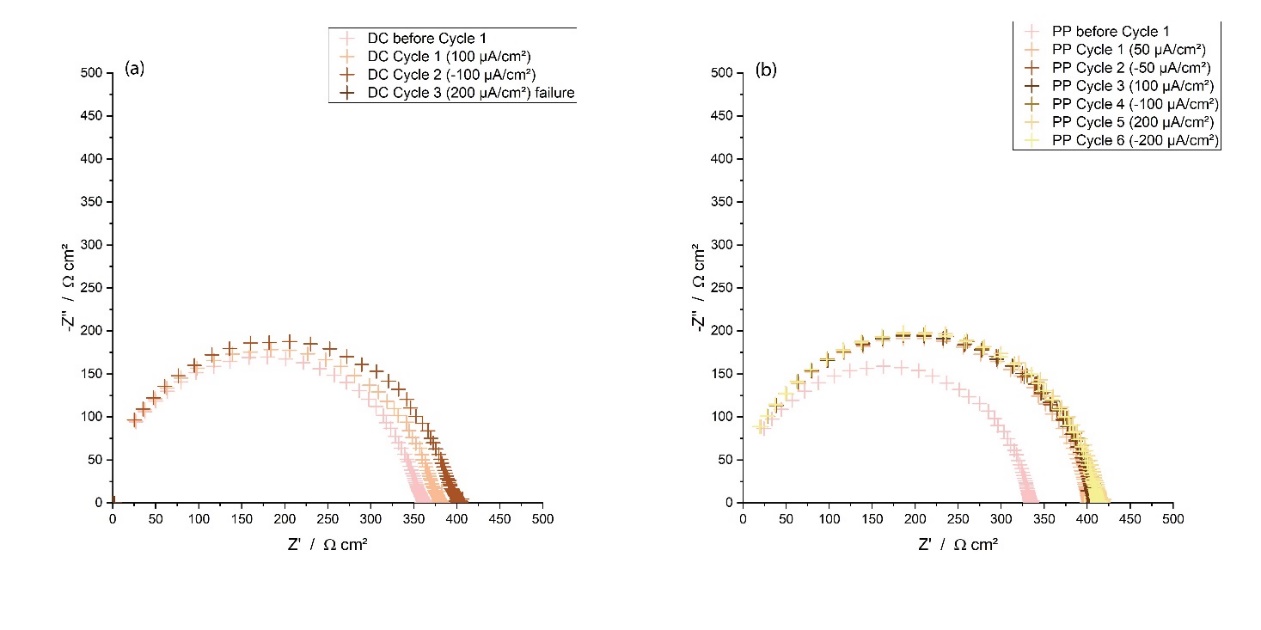


**Supplementary Fig. 20: EIS data of HP samples.** (a) Nyquist plot showing the impedance evolution of an HP sample cycled in a Li|HP|Li cell at 21±1°C with direct current (DC) until failure. (b Nyquist plot showing the impedance evolution of an HP sample cycled in a Li|HP|Li cell at 21±1°C with pulsed current application (PP) until the same current density as the sample in (a).


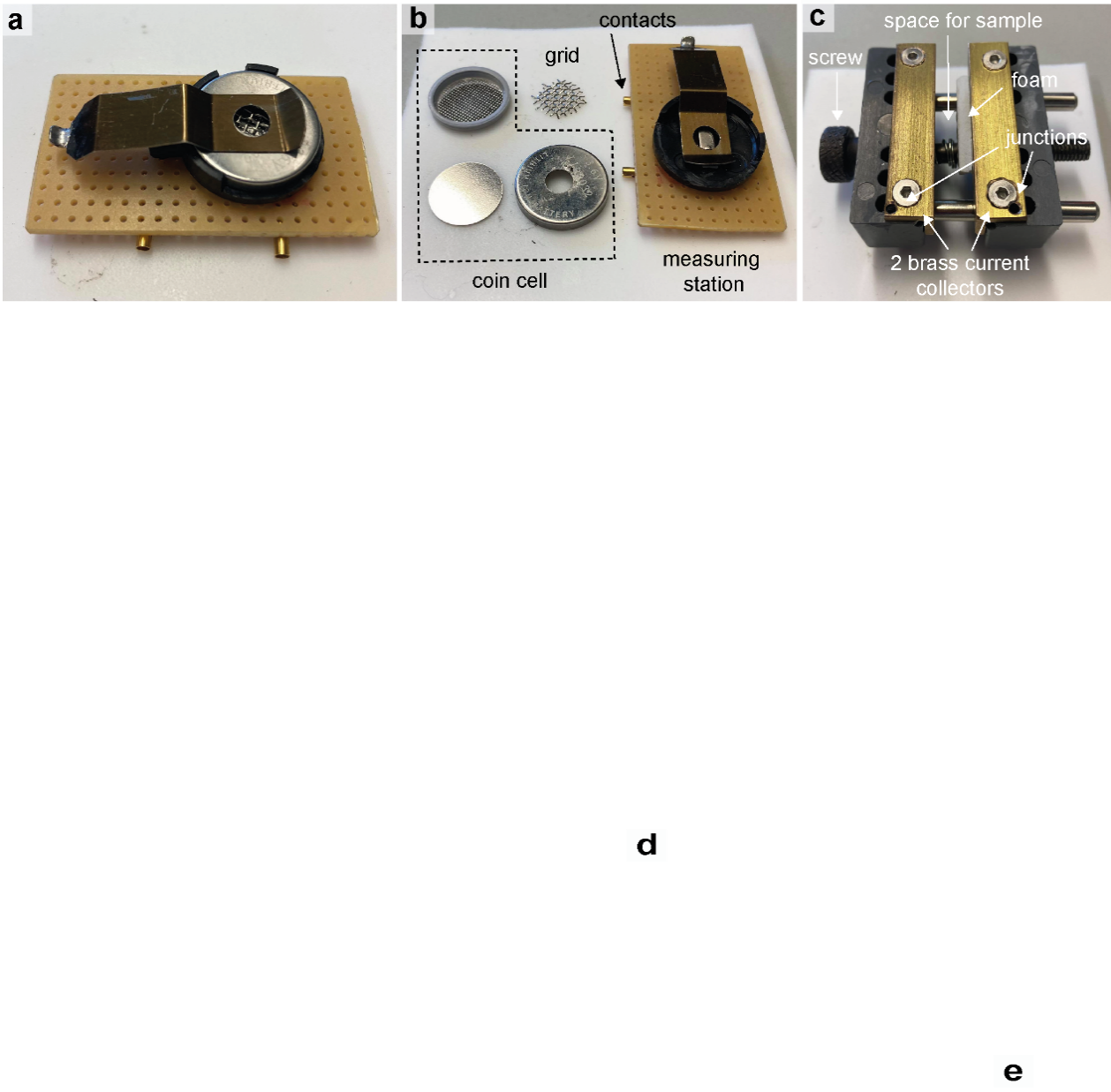


**Supplementary Fig.21:** **Self-assembled setups used for electrochemical characterization.**(a,b) Homemade setup for the plating experiments consisting of a measuring station and a coin cell that can hold a PC pellet and a top grid as current collector. (c) Measuring setup used for the cycling and efficiency experiments of the SC and HP samples consisting of two parallel aligned brass current collectors equipped with a junction and a white foam in the middle to help with filament tracking. A screw is used for adjusting the distance to the respective sample.

**Supplementary Tab. 1:** Listing of the different coating approaches pursued during this study along with the formulation used for preparation of the alloy, tested interlayers (carbon C or gold Au) and a final qualitative rating ranging from – (worst case), over ~ (satisfactory) to + (best case). While the Li: metal M alloys were applied via dipping the specimen into a molten bath, interlayers were applied prior by abrading a pure graphite crucible onto the sample or sputtering (Au target). The Li:Sn alloy (marked in blue) was finally chosen as the go to coating strategy for all later measurements.

| **approach** | **formulation** | **tested interlayers** | **rating** |
| --- | --- | --- | --- |
| Li | pure Li metal | C, Au | - |
| Li:Sn | Li metal + 30 wt% Sn powder | C | + |
| Li:Na | Li metal + 21.4 mol% Na metal | C | ~ |
| Li:Zn | Li metal + 5 mol% Zn powder | C | ~ |

**Supplementary Tab. 2:** Listing of PC samples treated with either direct or pulsed current conditions (ms and μs range) in a symmetric Li||Li cell along with their measured ASR and CCD values at 21±1°C. The effective CCD (CCD_eff_) represents the CCD after consideration of the capacity decrease and the step size taken in the cycling protocol is additionally given for uncertainty estimation.

| **sample** | **Conditions** | **ASR** | **CCD** | **CCD_eff_** | **step** |
| --- | --- | --- | --- | --- | --- |
|  |  | Ω cm^2^ | μA/cm^2^ | μA/cm^2^ | μA/cm^2^ |
| PC DC 1 | DC | * | 250 | 250 | 50 |
| PC DC 2 | DC | * | 20 | 20 | 5 |
| PC DC 2 | DC | * | 325 | 325 | 5 |
| PC PP 1 | 1:1 (ms) | 4.4 | 400 | 200 | 200 |
| PC PP 2 | 1:2 (ms) | 1.5 | 600 | 200 | 200 |
| PC PP 3 | 1:3 (ms) | 34.1 | 600 | 150 | 200 |
| PC PP 4 | 1:5 (ms) | 60.8 | 600 | 100 | 200 |
| PC PP 5 | 1:10 (ms) | 19.1 | 1200 | 109 | 200 |
| PC PP 6 | 1:1 (μs) | 6.6 | 1200 | 600 | 200 |
| PC PP 7 | 1:2 (μs) | 8.8 | 2000 | 667 | 200 |
| PC PP 8 | 1:2 (μs) | 8.7 | 800 | 267 | 200 |
| PC PP 9 | 1:3 (μs) | 10.5 | 600 | 150 | 200 |
| PC PP 10 | 1:3 (μs) | 10.3 | 1800 | 450 | 200 |

* no data available

**Supplementary Tab. 3:** Listing of SC samples treated with either direct or pulsed current conditions (μs range) in a symmetric Li||Li cell along with their measured ASR and CCD values at 21±1°C. The effective CCD (CCD_eff_) represents the CCD after consideration of the capacity decrease and the step size taken in the cycling protocol is additionally given for uncertainty estimation.

| **sample** | **conditions** | **ASR** | **CCD** | **CCD_eff_** | **step** |
| --- | --- | --- | --- | --- | --- |
|  |  | Ω cm^2^ | μA/cm^2^ | μA/cm^2^ | μA/cm^2^ |
| SC DC1 | DC | 140.3 | 250 | 250 | 250 |
| SC DC 2 | DC | 115.4 | 400 | 400 | 200 |
| SC DC 3 | DC | 44.9 | 400 | 400 | 200 |
| SC DC 4 | DC | - | 1250 | 1250 | 50 |
| SC DC 5 | DC | - | 800 | 800 | 50 |
| SC DC 6 | DC | - | 3900 | 3900 | 50 |
| SC DC 7 | DC | - | 4950 | 4950 | 50 |
| SC PP 1 | 1:1 (μs) | - | 3530 | 1765 | 100 |
| SC PP 2 | 1:1 (μs) | - | 1600 | 800 | 100 |
| SC PP 3 | 1:1 (μs) | - | 2340 | 1170 | 100 |
| SC PP 4 | 1:1 (μs) | - | 900 | 450 | 100 |
| SC PP 5 | 1:1 (μs) | - | 1100 | 550 | 100 |
| SC PP 6 | 1:1 (μs) | - | 4800 | 2400 | 100 |

**Supplementary Tab. 4:** Listing of HP samples treated with either direct or pulsed current conditions (μs range) in a symmetric Li||Li cell along with their measured ASR and CCD values at 21±1°C. The effective CCD (CCD_eff)_ represents the CCD after consideration of the capacity decrease and the step size taken in the cycling protocol is additionally given for uncertainty estimation.

| **sample** | **conditions** | **ASR** | **CCD** | **CCD_eff_** | **step** |
| --- | --- | --- | --- | --- | --- |
|  |  | Ω cm^2^ | μA/cm^2^ | μA/cm^2^ | μA/cm^2^ |
| HP DC 1 | DC | 704.1 | 200 | 200 | 100 |
| HP DC 2 | DC | 86.0 | 500 | 500 | 50 |
| HP DC 3 | DC | 51.4 | 650 | 650 | 50 |
| HP DC 4 | DC | - | 1050 | 1050 | 50 |
| HP DC 5 | DC | - | 400 | 400 | 50 |
| HP DC 6 | DC | - | 450 | 450 | 50 |
| HP PP 1 | 1:1 (μs) | * | 2100 | 1050 | 100 |
| HP PP 2 | 1:1 (μs) | - | 6600 | 3300 | 100 |
| HP PP 3 | 1:1 (μs) | - | 1350 | 675 | 100 |
| HP PP 4 | 1:1 (μs) | - | 2750 | 1375 | 100 |
| HP PP 5 | 1:1 (μs) | - | 3200 | 1600 | 100 |
| HP PP 6 | 1:1 (μs) | - | 2100 | 1050 | 100 |
| HP PP 7 | 1:1 (μs) | - | 4800 | 2400 | 100 |

* no data available

**Supplementary Tab. 5:** List of all CCD values found in literature for LLZO being above 1 mA/cm^2^. Besides the stoichiometric composition, also the category of improvement (alloy, additive, interface engineering) as well as the cycling conditions (T, p) and pellet quality (density, ASR) are given if mentioned in literature. Rows marked in red are measurements carried out at an elevated temperature and/or pressure and are therefore unfit for direct comparison to the rest of the CCD values.

| **CCD** | **composition** | **additive** | **alloy** | **interface** | **T** | **p** | **density** | **ASR** | **Ref** |
| --- | --- | --- | --- | --- | --- | --- | --- | --- | --- |
| mA/cm² |  |  |  |  | °C |  | % | Ω cm^2^ |  |
| 1.1 | Li_6.5_La_3_Zr_1.5_Ta_0.5_O_12_ | - | - | PbO/ZnO/ Co_3_O_4_ | RT | - | - | 10 | 1 |
| 1.1 | Li_6.4_La_3_Zr_1.4_Ta_0.6_O_12_ | - | - | CoO/Li_2_O | 25 | ? | 95 | 12.3 | 2 |
| 1.15 | Li_6.4_La_3_Zr_1.4_Ta_0.6_O_12_ | 15 wt% excess LiOH·H_2_O | - | Li_x_Sn:Li_2_O | RT | 10 N cm^−2^ | - | 25 | 3 |
| 1.15 | Li_6.4_La_3_Zr_1.4_Ta_0.6_O_12_ | excess lithium | - | Au | 25 | - | ∼96% | 3 | 4 |
| 1.2 | Li_6.5_La_3_Zr_1.5_Ta_0.5_O_12_ | - | - | SnS_2_ | 100 | Swagelok | - | 17 | 5 |
| 1.2 | Li_6.5_La_3_Zr_1.5_Nb_0.5_O_12_ | Li_3_PO_4_ + 10 wt% excess LiOH.H_2_O | - | - | RT | - | 90.9 | - | 6 |
| 1.3 | Li_6.5_La_3_Zr_1.5_Ta_0.5_O_12_ | - | Sr:Li | - | RT | - | - | - | 7 |
| 1.4 | Ta-doped LLZO | excess LiOH·H_2_O | O_V_-ZnO | - | RT | - | - | 55 | 8 |
| 1.4 | Li_6.75_La_3_Zr_1.75_Ta_0.25_O_12_ | - | - | Pt | RT | coin cell | - | 9 | 9 |
| 1.4 | Li_6.5_La_3_Zr_1.5_Ta_0.5_O_12_ | Li_6_Zr_2_O_7_ | - | Au | 25 | coin cell | 97.21 | 1.65 | 10 |
| 1.7 | Li_6.4_La_3_Zr_1.4_Ta_0.6_O_12_ | - | Li:Mo  (α-MoO_3_ nanobelts) | - | RT | - | - | 1 | 11 |
| 1.7 | Li_6.4_La_3_Zr_1.4_Ta_0.6_O_12_ (nm-sized) | Al_2_O_3_ | - | Au | 60 | Micro-clamp | 98.5 | 129.4 | 12 |
| 1.8 | Li_6.5_La_3_Zr_1.5_Ta_0.5_O_12_ | - | - | LiF:LiCl | 25 | - | >96 | 110 | 13 |
| 1.95 | Li_6.5_La_3_Zr_1.5_Ta_0.5_O_12_ | 4 wt% MgO | - | Au | 25 | - | 97.9 | 18 | 14 |
| 2 | Li_6.4_La_3_Zr_1.4_Ta_0.6_O_12_ | - | - | PZL | RT | 30 MPa | - | 1.9 | 15 |
| 2.2 | Li_6.5_La_3_Zr_1.5_Ta_0.5_O_12_ | - | - | MoS_2_ | 100 | - | - | 14 | 16 |
| 2.8 | Li_6.5_La_3_Zr_1.5_Ta_0.5_O_12_ | - | - | Cu doped Li_3_Zn | 28 | coin cell | 98 | 30 | 17 |
| 3.8 | Li_6.25_Al_0.25_La_3_Zr_2_O_12_  Li_6.5_Al_0.15_La_3_Zr_1.5_Ta_0.5_O_12_ | - | - | Au | 40 | 3.3 MPa | >99 | 9–15 | 18 |
| 6 |  |  |  |  | 60 | 3.3 MPa | >99 | 9–15 | 18 |
| 4 | Li_6.4_La_3_Zr_1.4_Ta_0.6_O_12_ | - | - | ZNR | RT | - | - | - | 19 |
| 6.5 | Li_6.4_La_3_Zr_1.4_Ta_0.6_O_12_ | - | Li:Sn | - | 21 ± 1 | <(1.8 ± 0.8) kPa | >99 | - | This study |
| 10 | Li_6.75_La_2.75_Ca_0.25_Zr_1.5_Nb_0.5_O_12_ | - | - |  | RT | button cell | >99 | 7 | 20 |

**Supplementary Note 1: Ion conductivity and area specific resistance**

Supplementary Fig. 5 and Supplementary Fig. 6 show the Electrochemical Impedance Spectroscopy (EIS) data of all the Single Crystalline (SC) and Hot-Pressed (HP) LLZO samples tested herein, once in form of a Nyquist (a) and once in form of a Bode (b) plot. For the SC samples, only one semicircle in the Nyquist plot is visible, with one corresponding plateau in the Bode plot. Due to the lack of grain boundaries in a single crystal this contribution can be assigned to the bulk. Except for the first ones, HP DC1 and 2, the HP samples show a similar behavior and only reveal one contribution in either plot. HP DC1 was not heat-treated and HP DC2 did not undergo a polishing step directly prior to coating. In both cases, an additional contribution connected to the electrode|electrolyte interface is present in the plots and results in a much higher Area Specific Resistance (ASR, see **Supplementary Tab. 4**) as opposed to the treated samples.

**Supplementary Note 2: Applied pressure determination**

In our homemade setup a spring holds the vice jaw in place counteracting the force exerted from the screw. The spring constant of this spring (2,5 ± 0,5 N/m) was determined by measuring the compression at two different force levels (15 and 30 N) at 25°C. The force exerted by turning the screw one turn was determined to be (3.3 ± 0.5) × 10^-4^ N. Since the sample was fixed by turning the screw for one turn after the jaws contact it, the pressure can be calculated from the sample geometry (1.84 × 10^-6^ m²). This leads to a possible pressure on the sample of (1.8 ± 0.8) kPa.

**Supplementary Note 3: Preliminary cycling experiments and identification of favorable protocol**

Overall, a typical pulsing sequence in this study is composed of an A:B period, with A representing the current application duration in relation to the pausing time B. In case of a 1:1 sequence, this would mean, that the pausing time matches the current application time, whereas in a 1:2 sequence the pausing time is double the current application time. In the ms-range, A was set to 2 ms (due to instrumental limitations) whereas in the μs-regime, all experiments were conducted with a 1 μs current application time. Overall, first pulsing tests with the PC-samples in the ms-regime revealed no improvement of the electrochemical operation in contrast to direct current application. Reducing the time scale to the μs-range, however, lead to overall significantly higher CCD values (see Supplementary Tab. 2). As evident from the reached CCD values, a low ASR value does not necessarily guarantee high electrochemical performance. This becomes especially evident for sample PC PP 7 and 8 which were both equally treated with a 1:2 pulsing sequence and showed similar ASR values of the pristine pellets (8.8 Ωcm^2^ vs. 8.7 Ωcm^2^). Whereas PC PP 7 reached an effective CCD of 667 μA/cm^2^, PC PP 8 already shorted at 267 μA/cm^2^. While the ASR value is highly useful in predicting the quality of the SSE wetting, it does not necessarily reflect the occurrence of surface scratches, which, at high current densities, can become the nucleation sites for Li filaments. Overall, the cycling experiments showed that shorter pausing times appear to be most favorable, which is why all further PP experiments with the SC and HP samples were conducted with a 1:1 μs sequence.

**Supplementary Note 4: Failure identification**

Examples of a typical symmetric Li||Li cell with SC and HP electrolytes sample are shown in Supplementary Fig. 9 and Supplementary Fig. 10, respectively. Due to simultaneous optical tracking during electrochemical operation, failure identification was simplified, and it could be observed that any significant potential drop was related to Li filament formation. The whole cycling profile in general is composed of the overall cycling curve along with a close-up window showing the voltage drop associated with failure and the corresponding optical evaluation ranging from the intact sample and initiation of the Li filament down to the propagation route and final short circuit. In both cases, the voltage profile shows a significant drop in potential once Li starts to deposit at the right interface and decreases as the dendrite makes its way through the SSE. Once it reaches the opposing side the potential drops to zero. In some cases, filaments would not grow through the entire SSE or cause the voltage to fall to zero.

**Supplementary Note 5: CCD studies of SC and HP samples**

Supplementary Tab. 3 and Supplementary Tab. 4 contain a list of the SC and HP samples along with their cycling program, interfacial resistances prior to cycling as well as the CCD and effective CCD_eff_ values. The CCD value was chosen to be the current density value where a characteristic potential drop was starting. Additionally, the step size of the program is listed to allow for an uncertainty approximation of the achieved electrochemical performances. The CCD value was therefore specified in the main text as “maximum value – current step size of cycling program”, as the actual CCD lies within this current density range. In case of the SC samples, the first direct current study (SC DC 1-3) was carried out with samples of lower quality, which were cut with a diamond saw and simply sanded and polished with SiC paper like the PC samples. In comparison to the shaped and polished specimens a much higher interfacial resistance could be determined, and the CCD values were lower. In case of the HP samples, the impact of a missing heat treatment and final polishing step is demonstrated in the case of sample HP DC 1. Compared to the SC samples however, the difference does not appear to be as prominent.

**Supplementary Note 6: Proof of concept**

To check on the effectiveness of pulsed currents in the μs-regime, efficiency and plating experiments were conducted with the PC samples. In case of the plating experiments an asymmetric cell employing a Li and Au electrode was employed and Li metal was plated for a total time of 4 hours with a 1:1 (μs) pulsing sequence. The deposition process was then qualitatively examined in-situ via Optical Microscopy by focusing through the grid of the Au electrode current collector. Supplementary Fig. 7 shows the complete cycling profile along with a close-up of the voltage sequence and an image of the pristine PC surface (1), as well as one picture after finishing the plating (2) and subsequent stripping step (3). Image 2 clearly shows that pulse plating in the μs-range is able to transfer Li across the SSE to the opposing electrode. It should be noted that the surface morphology in this case does not transfer to the situation in the typical symmetric cells used for the regular cycling experiments.

The efficiency of this plating process was then checked by depositing Li metal via 1:1 μs pulsed currents for 4 hours and subsequently stripping it again with direct current application for half the time, being 2 hours in this case. The assumption is that if pulsed currents were less effective than direct current at Li transfer, then the time to remove the same amount of Li would deviate significantly. Despite minor voltage plateaus, no sharp voltage increase was detected during this 2 hour time frame, which is why it can be assumed that the efficiency of pulsed currents at this time scale is close to 100 %.

**Supplementary Note 7: Reversibility of lithium filaments**

Throughout the whole cycling study of the SC samples, one phenomenon that was encountered several times was the reversibility of lithium filaments after the cell was shorted. As shown in Supplementary Fig. 12 shortly after the beginning of the second half-cycle, the SSE is completely penetrated by Li filaments and the voltage drops to around 0 V. In the subsequent half-cycle, however, this process is reversed as the filaments form back and the sample appears to be optically and electrochemically intact again. Typically, the samples would then short-circuit in the subsequent half-cycle once again and remain in this state for good. Supplementary Fig. 13 shows the evolution of the Electrochemical Impedance Spectroscopy response during such a short-circuiting event where measurements were taken in between half-cycles. Prior to Li filament formation a constant impedance response was obtained (grey markers) up to the last measurement prior to failure (green markers). Once the Li filaments connect both electrodes, a typical short-circuit behavior is recorded (light red) until in the subsequent half-cycle the filaments reverse, and an impedance response is measured again (dark red).

**Supplementary Note 8: Modeling stress buildup under pulsed conditions**

In assessing whether pulse plating could mitigate stress buildup in lithium filaments, the authors used COMSOL Multiphysics finite element analysis to model the deformation of an expanding metal filament (as discussed in the Methods). Because fracture represents the release of stored elastic energy, the value of the elastic energy stored under a given set of boundary conditions should track with the propensity for fracture. Furthermore, due to force balance at the metal electrolyte interface, the elastic energy stored within the electrolyte should track with the elastic energy stored in the metal filament. Thus, the stored elastic energy in a metal filament can serve as a proxy for the propensity of the electrolyte surrounding the filament to fracture.

Pulse plating was simulated under four conditions: (1) application of 1 mA/cm^2^ direct current for 0.5 seconds followed by 0.5 seconds pause (no current application), (2) 0.5 mA/cm^2^ direct current for 1 second followed by 1 second pause, (3) 0.25 mA/cm^2^ direct current for 2 seconds followed by 2 seconds pause, and (4) 0.5 mA /cm^2^ “effectively” pulsed direct current (repeated segments of 1 µs of 1 mA/cm^2^ current followed by 1 µs of no current) over a total time of 1 second. Supplementary Fig. 15 displays the resulting elastic energy stored in the filament versus normalized time (that is, time / 2 for conditions (2) and (4), and time / 4 for condition (3)).

Because the time displayed on the x-axis is normalized by the total plating time for simulations of equal plated capacities, the x-axis represents a measure of plated capacity during the simulation. At a given value on the x-axis, all four plating simulations possess an identical volume of metal “plated.” As seen in this figure, the elastic energy stored in the filament during plating increases with increased direct current density during the plating segment. However, the 0.5 mA/cm^2^ “effective” current density from the pulse plating simulation possesses a similar elastic energy stored to the 0.5 mA/cm^2^ conducted under direct current plating. Thus, mechanical relaxation of stresses within the filament that may occur during the “off” segment of pulse plating does not appear to substantially mitigate the electrolyte fracture as compared to the direct current plating case.

**Supplementary Note 9: The role of the defect shape**

Consider a LLZTO surface that is composed of different sized defects, such as grain boundaries, flaws or pores in contact with Li metal. Once such a defect is filled up with Li and a high enough current is applied such that the Li-ion flux towards the tip exceeds the Li-ion flux from the tip, a significant stress builds up and eventually fractures the ceramics. Our previous study found filaments to become critical after they exceed an aspect ratio of ≈ 3:1.^21^ For a simple analysis the stress distribution is assumed constant so that textbook fracture mechanics apply. When the applied stress intensity factor $K_{I}$ is larger than the critical stress intensity factor $K_{Ic}$ (i.e., the fracture toughness), a crack nucleates and propagates. $K_{Ic}$ can be calculated by considering the applied stress $\sigma$, a geometry factor $Y$ (which is reasonably close to 1) and the crack length $c$ using Eq. 5.

$K_{Ic}=\sigma Y*\sqrt{c}$ (5)

Supplementary Fig. 16 shows the dependence of the generated stress intensity factor on the crack length at an assumed homogeneous internal stress of 500 MPa, based on Eq 5, (note: arbitrary number since this value is not known for LLZO so far) is shown. The $K_{Ic}$of a grain and grain boundary can be assumed as 0.7 MPa m^0.5^ and 0.3 MPa m^0.5^, respectively.^22^ In this simplified model, the grain boundary and bulk defect must be larger than about 300 nm and 2 µm, respectively, to generate a stress intensity factor sufficiently high to advance a crack on a pre-existing flaw. In consequence, there is a certain minimum flaw size for each current density, under which no filament generation occurs. However, before a flaw can become critical, it must be filled with Li which will require a certain amount of time depending on the applied current density, as plotted in Fig 6**d**. Therefore, smaller flaws can be filled and, hence, become critical faster than larger ones. After becoming critical the propagation speed of the flaw is much higher than the filling speed making it the main failure cause. While the possible minimum flaw size in a single crystal is only determined by the surface preparation, grains and grain boundaries mark the smallest possible non removable flaws in a polycrystal. Hence it seems logical for the HP sample to fail earlier.

**Supplementary Note 10: Active surface area comparison**

Two HP samples were prepared and coated with Li. One was cycled until failure with direct current application, failing at 200 µA/cm², whereas the other one was cycled with pulsed currents and stopped at 200 µA/cm² without failure. The interfaces of both samples, where lithium was plated, were then investigated via Focused Ion Beam-Scanning Electron Microscopy, as shown in Supplementary Fig. 19. For both samples an intimate contact between the LLZTO and the Li electrode is seen. There is also no discernible difference in the apparent contact area, as a few occluded voids are close to the interface region in both cases. The evolution of the impedance for both samples is shown in Supplementary Fig. 20.

REFERENCES

1. Cai, M. et al. Robust Conversion-Type Li/Garnet interphases from metal salt solutions. *Chem. Eng. J.* **417,** 129158 (2021).

2. Lu, G. et al. Universal lithiophilic interfacial layers towards dendrite-free lithium anodes for solid-state lithium-metal batteries. *Sci. Bull.* **66.17,** 1746-1753 (2021).

3. Chen, Y. et al. Nanocomposite intermediate layers formed by conversion reaction of SnO_2_ for Li/garnet/Li cycle stability *J. Power Sources* **420,** 15-21 (2019).

4. Huang, X. et al. None-mother-powder method to prepare dense Li-garnet solid electrolytes with high critical current density. *ACS Appl. Energy Mater.* **1.10,** 5355-5365 (2018).

5. Zhou, D. et al. Garnet electrolytes with ultralow interfacial resistance by SnS2 coating for dendrite-free all-solid-state batteries. *ACS Appl. Energy Mater.* **4.3,** 2873-2880 (2021).

6. Yang, L., et al. Rapid preparation and performances of garnet electrolyte with sintering aids for solid-state Li–S battery. *Ceram* **47.13,** 18196-18204 (2021).

7. He, X. et al. Tuning Interface Lithiophobicity for Lithium Metal Solid-State Batteries. *ACS Energy Letters* **7.1,** 131-139 (2021).

8. Wei, Y. et al. An oxygen vacancy-rich ZnO layer on garnet electrolyte enables dendrite-free solid state lithium metal batteries. *Chem. Eng. J.***433,** 133665 (2022).

9. Liao, Y.-K., et al. Extensively Reducing Interfacial Resistance by the Ultrathin Pt Layer between the Garnet-Type Solid-State Electrolyte and Li–Metal Anode. *ACS Appl. Mater. Interfaces* **13.47,** 56181-56190 (2021).

10. Zheng, C. et al. Grain boundary modification in garnet electrolyte to suppress lithium dendrite growth. *Chem. Eng. J.* **411,** 128508 (2021).

11. Liu, B. et al. A simple strategy that may effectively tackle the anode-electrolyte interface issues in solid-state lithium metal batteries. *Chem. Eng. J.* **427,** 131001 (2022).

12. Huang, Z. et al. Enhanced performance of Li_6.4_La_3_Zr_1.4_Ta_0.6_O_12_ solid electrolyte by the regulation of grain and grain boundary phases. *ACS Appl. Mater. Interfaces* **12.50,** 56118-56125 (2020).

13. Ruan, Y. et al. A 3D cross‐linking lithiophilic and electronically insulating interfacial engineering for garnet‐type solid‐state lithium batteries. *Adv. Funct. Mater.* **31.5,** 2007815 (2021).

14. Guo, H. et al. Achieving high critical current density in Ta-doped Li_7_La_3_Zr_2_O_12_/MgO composite electrolytes. *J. Alloys Compd.* **856,** 157222 (2021).

15. Wan, Z. et al. Three-dimensional alloy interface between Li_6.4_La_3_Zr_1.4_Ta_0.6_O_12_ and Li metal to achieve excellent cycling stability of all-solid-state battery. *J. Power Sources* **505,** 230062 (2021).

16. Fu, J. et al. In situ formation of a bifunctional interlayer enabled by a conversion reaction to initiatively prevent lithium dendrites in a garnet solid electrolyte. *Energy Environ. Sci.* **12.4,** 1404-1412 (2019).

17. He, X, et al. Cu-Doped Alloy Layer Guiding Uniform Li Deposition on a Li–LLZO Interface under High Current Density. *ACS Appl. Mater. Interfaces* **13.35,** 42212-42219 (2021).

18. Taylor, N. J. et al. Demonstration of high current densities and extended cycling in the garnet Li_7_La_3_Zr_2_O_12_ solid electrolyte. *J. Power Sources* **396,** 314-318 (2018).

19. Zhong, Y. et al. A Highly Efficient All‐Solid‐State Lithium/Electrolyte Interface Induced by an Energetic Reaction. *Angew. Chem. Int. Ed* **59.33,** 14003-14008 (2020).

20. Hitz, G. T. et al. High-rate lithium cycling in a scalable trilayer Li-garnet-electrolyte architecture. *Mater. Today* **22,** 50-57 (2019).

21. Porz, L. et al. Mechanism of lithium metal penetration through inorganic solid electrolytes. *Adv. Energy Mater.* **7.20,** 1701003 (2017).

22. Porz, L. et al. Characterizing brittle fracture by modeling crack deflection angles from the microstructure. *J. Am. Ceram. Soc.* **98.12,** 3690-3698 (2015).
