# Supplementary material for: Effect of pulse-current-based protocols on the lithium dendrite formation and evolution in all-solid-state batteries
Source: Nat Commun. 2023 Apr 27;14:2432. doi: 10.1038/s41467-023-37476-y (PMC10140044; doi:10.1038/s41467-023-37476-y)
Supplement: Supplementary file 3 — Description of Additional Supplementary Files [file 41467_2023_37476_MOESM3_ESM.pdf]

Description of Additional Supplementary Files

Title: Supplementary Movie 1

Description: Straight Li propagation in SC sample from intact condition to short circuit.

Title: Supplementary Movie 2

Description: Branched Li propagation in SC sample from intact condition to short circuit.

Title: Supplementary Movie 3

Description: Diffuse Li propagation in SC sample from intact condition to short circuit.

Title: Supplementary Movie 4:

Description: Reversible Li propagation in an SC sample.

Title: Supplementary Movie 5

Description: Li propagation in an HP sample from intact condition to short circuit.
